# Using Eye Tracking to Assess Reading Performance in Patients with Glaucoma: A Within-Person Study

**DOI:** 10.1155/2014/120528

**Published:** 2014-05-05

**Authors:** Nicholas D. Smith, Fiona C. Glen, Vera M. Mönter, David P. Crabb

**Affiliations:** Division of Optometry and Visual Science, School of Health Sciences, City University London, London EC1V 0HB, UK

## Abstract

Reading is often cited as a demanding task for patients with glaucomatous visual field (VF) loss, yet reading speed varies widely between patients and does not appear to be predicted by standard visual function measures. This *within-person* study aimed to investigate reading duration and eye movements when reading short passages of text in a patient's worse eye (most VF damage) when compared to their better eye (least VF damage). Reading duration and saccade rate were significantly different on average in the worse eye when compared to the better eye (*P* < 0.001) in 14 patients with glaucoma that had median (interquartile range) between-eye difference in mean deviation (MD; a standard clinical measure for VF loss) of 9.8 (8.3 to 14.8) dB; differences were not related to the size of the difference in MD between eyes. Patients with a more pronounced effect of longer reading duration on their worse eye made a larger proportion of “regressions” (backward saccades) and “unknown” EMs (not adhering to expected reading patterns) when reading with the worse eye when compared to the better eye. A between-eye study in patients with asymmetric disease, coupled with eye tracking, provides a useful experimental design for exploring reading performance in glaucoma.

## 1. Introduction

Glaucoma is a leading cause of visual impairment and affects a significant number of the elderly populations [1]. The conventional view of vision loss in glaucoma suggests disruption of peripheral vision and minimal impact on tasks that require good central vision, like reading. However, patients with glaucoma regularly self-report difficulties with reading [2–6]. Furthermore, evidence is emerging from experimental studies showing that some patients with glaucoma have impaired reading performance when compared to their visually healthy peers. These impairments are particularly evident for patients with advanced or bilateral visual field loss [7–9] when reading small size text [10]; when reading text at low contrast [11]; or when reading for sustained periods of time [12]. However, not all patients displayed reduced reading speeds in these studies, with some patients appearing to be much more affected than others. A limiting feature of the studies that have generated these results is that reading speed, as an experimental outcome measure, is subject to much between-person variability: it is very difficult to isolate the impact of the glaucomatous visual field loss from all the other factors, such as age, visual acuity, and cognitive and reading ability, that might contribute to slower reading. Furthermore, differences in eye movement patterns may also influence reading speed. Eye movements supplement information about how long a person takes to read, by giving insight into* how *they are reading. Previous research has considered eye movements in patients with glaucoma compared to visually healthy controls when carrying out a number of other visual tasks, such as visual search [13], face recognition [14], viewing of photographs [15], and watching of driving videos [16]. In these studies, patients sometimes displayed different eye movement patterns on average to controls, although it was suggested that some patients may “adapt” their eye movements in ways that enable them to function better in the task [13, 14]. However, the case-control design that featured in all these studies again made it difficult to discern the nature of the contribution of visual field loss to changes in eye movement behaviour.

As yet no studies have considered performing a* within-person*, or between-eye, reading study to examine the impact of glaucomatous visual field loss on reading performance: the idea here would be that a more damaged eye could be compared with a less affected fellow eye. An experimental design such as this might proffer advantages over studies comparing patients to controls, where large numbers of people are needed to demonstrate effects. In addition, experimental studies of reading speed in glaucoma have been constrained to those where reading “out loud” or timed silent reading is simply the main, or only, outcome measure. One recent study incorporated eye tracking when investigating reading performance in glaucoma [17]: the findings of that case-control study, which measured the maximum and minimum sizes of eye movements made during a reading task by patients compared to controls, hinted that glaucoma may lead to some alterations in fixation behaviour. However, to date, no studies have used an eye tracker to measure more task-specific saccades (i.e., rapid eye movements occurring between locations on the text) to tease out the effects that might result from glaucomatous visual field loss whilst reading short passages of text.

In this study, we explore the usefulness of comparing monocular reading performance in patients with asymmetric glaucomatous visual field loss. The study measures reading performance using eye tracking whilst participants silently read very short passages of text. Our main hypothesis is that patients will take longer to read short passages of text in what is considered to be their worse eye (most visual field damage) when compared to their better eye (least visual field damage); we aim to do this in just a small sample of patients in order to demonstrate the effectiveness of the experimental design. We also, as a secondary aim, test the idea of determining different types of reading-specific saccadic eye movements, in an automated fashion, specifically eye movements that occur in a forward direction (forward saccades), saccades that “backtrack” over previously read text (regressions), those that occur between the end of one line and the beginning of the next (line change saccades), and eye movements that do not fit expected patterns (unknown saccades). Next we investigate if any of these measurements from this automated approach are associated with the size of between-eye deficits in standard measures of visual function.

## 2. Methods

Participants were recruited from a database of patients that had taken part in previous studies conducted at City University London [13, 18]. All patients had a clinical diagnosis of primary open angle glaucoma and had no other ocular diseases. Patients were contacted if they had previously presented with asymmetric visual field loss between eyes as measured using a central 24-2 SITA Standard Test on the Humphrey Visual Field Analyzer (HFA, Carl Zeiss Meditec, CA, USA). This was quantified by considering the HFA mean deviation (MD); this summary measure expresses the average reduction in the visual field relative to a group of visually healthy age-matched observers [19]. Participants were only invited to the study if the MD differed by more than 6 dB between eyes. This value represents a clinically significant difference as used in staging schemes for visual field severity [20].

The study was approved by the Ethics Committee for the School of Health Sciences, City University London. All participants gave their informed consent and the study conformed to the Declaration of Helsinki.

### 2.1. Standard Vision Testing

Fourteen patients were recruited and all testing was carried out on one day. Visual acuity (VA) as measured with the Early treatment diabetic retinopathy study (ETDRS) chart and contrast sensitivity as measured with the Pelli-Robson chart (PR Log CS) were assessed monocularly. Astigmatic error was less than ±2.5 dioptres in all those recruited. Visual field tests (central 24-2 and 10-2 SITA Standard) were conducted in each eye using a HFA. On testing (central 24-2), two of the 14 patients had a between-eye MD difference of less than 6 dB (4.7 and 4.8 dB). We decided that these patients should still be included in the study. From this point we define the patient's eye with the worse VF damage (worse MD) to be the “worse eye” and the fellow eye to be the “better eye.”

The reading experiment was performed on a 56 cm CRT computer monitor displaying at a resolution of 1600 by 1200 pixels and a refresh rate of 100 Hz (Iiyama Vision Master PRO 514, Iiyama Corporation, Tokyo, Japan). Participants were seated (with a head rest) in front of the computer screen. Each participant was fitted with a set of trial frames with the appropriate refractive correction. One eye was randomly selected and then occluded by inserting a blackout lens into the trial frames. Participants were then presented with 50 different texts (trials) on the screen, one at a time, and were asked to silently read them “as quickly and accurately as possible.” Once the participants had read the 50 texts, they had a short break before repeating the task using their alternate eye with 50 novel texts. Participants read the same 100 texts but in a randomised order. Each text consisted of one sentence, distributed over two lines, using the “Courier New” font at size 38 in which each letter subtended a maximum height of 0.75° visual angle and a constant width of 0.6°. The standardised passages of text had an average Flesch-Kincaid readability score of 4.6 and were the same as those used by Kabanarou and Rubin [21]. The background brightness was 33.4 cd/m^2^ and the text was displayed at 0.04 cd/m^2^. Each paragraph subtended 21° width and 3° in height.

Eye movements were recorded simultaneously during the reading task using an EyeLink 1000 (SR Research Ltd., Mississauga, Ontario, Canada) which was set to record the participant's eye location at 1000 Hz. It is claimed that the EyeLink 1000 measures at an average accuracy of better than 0.5°. The saccade detection thresholds were defined by a velocity greater than 30°/s and acceleration above 8000°/s^2^. Before the study commenced, a calibration was performed and had to be classified as a “good” standard as set by the instrument. Furthermore, between each trial (each displayed sentence) a drift check was performed and, if a substantial drift had occurred, a recalibration would be carried out.

### 2.2. Analysis of Eye-Tracking Data

To prepare the eye movement data for analysis, we developed a novel preprocessing technique. These methods adjusted for calibration errors in the eye tracking and ensured that only those saccades relevant to the reading task were included. Secondly, we report a novel method of classifying reading-specific eye movements according to their saccade type, that is, whether they occurred from left to right (forward saccade), right to left (regression), or between lines (line change) or did not conform to expected reading patterns (unknown saccade). We report both of these methods here as they may be relevant to other studies using eye tracking to measure reading performance. Note that the techniques described below do not require information about the specific content of the underlying text, such as details of the words and characters, but only the locations of the start and end of the text.

### 2.3. Preprocessing

**Figure 1:**
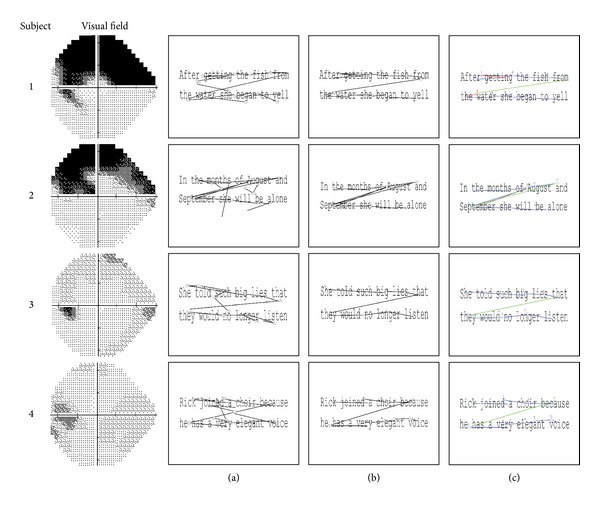
Four examples of scanpaths from four different glaucoma patients with their visual fields on the left. The start and end of each saccade are represented by a circle. Column (a) shows the original scanpaths made by the four participants reading the text. Column (b) shows the scanpath after the rotation has been corrected and reading-specific saccades have been extracted using the preprocessing algorithm. Column (c) shows the scanpath results from the clustering and classification algorithm. The number represents the order in which the saccades occurred, and the colours represent the classification that was attributed to them by the automated clustering algorithm (blue: forward saccade, green: between line saccade, red: regression, and brown: unknown).

Data from the eye tracker was used to determine reading duration for each trial in addition to identifying the key eye movement patterns made whilst reading the texts. The eye tracker was running before the display of each text in order to ensure that all eye movements were recorded, meaning that it was highly likely that some additional eye movements were made prior to beginning to read each sentence that were irrelevant to the task. Furthermore, the drift correction carried out before each trial meant that the participant always began the trial by fixating in the middle of the screen, therefore introducing bias into subsequent eye movement recordings. It was therefore necessary to pinpoint the exact points at which the person actually began reading the sentence and the point at which they finished reading. Use of an automatic real-time start and end point has the potential to misidentify when the person started or finished reading, as this technique uses fixed points on the screen and therefore assumes perfect calibration of the eye tracker. To address this issue, a novel “preprocessing” method was therefore implemented and is reported in detail here because it may be of use in other eye-tracking experiments. Some examples of preprocessed scanpaths are shown in Figure 1(a), showing additional saccades that occurred before and after the patient read the passage.

The first stage of the preprocessing algorithm attempted to correct any rotational errors in the eye movement data. As the text was displayed centrally, small errors in edge calibration were not of huge concern for this particular task; however inspection of scanpaths revealed that data sometimes appeared to be rotated along the centre. To correct this, it was assumed that all small saccades running ±20° along the horizontal (approximation of reading between words) should be corrected to correspond with the angle of the text (average angle of horizontal or 0°). Therefore, the circular median of all these ±20° angles of the saccades was calculated per trial, and all saccades were rotated (corrected) by this amount. Visual analysis of scanpaths also confirmed that, on being first presented with a text, participants sometimes made several involuntary eye movements at locations on the screen that were irrelevant to the task itself, before adjusting their gaze position so that they could start reading from the beginning of the sentence. In order that the analysis would only include those eye movements that were relevant to the task, an automated procedure was developed that determined which eye movements coincided with the text's start and end location, thereby filtering out all other irrelevant eye movements. This process involved a series of steps to identify the start and end point locations signalling the start and end point of reading each text. The standard preset SR Research EyeLink parser (edf2asc) results in sharp downward movements being recorded at the point just before the pupil disappears (i.e., during a blink). Sharp downward saccades do not correspond with reading, so these were identified and excluded specifically any saccade with an amplitude >6° and with an angle of between 250° and 290°. Next, we aimed to detect the starting point of the saccade nearest to the first word of the text and the end point of the saccade closest to the final word of the text. However, this procedure was complicated by the fact that the text was rectangular in shape, with the height being substantially smaller in size than the width, a factor that would bias end point detection. For instance, the end point of a saccade made at the end of the first line of text (i.e., top right of the text) could be incorrectly classified as being nearer to the end of the text than a saccade made on the line below. We therefore normalised the locations of the saccade start and end points in order to make the axes equal. Specifically, the Euclidian distance from (0, 0) (top left) for each saccade start point and the distance from (1, 1) (bottom right) for each saccade end point were calculated, creating two sets of distances. An exponential weighting was applied to these two sets of distances. As such, the more the distance value increases the further the point is from the start location. The start saccade was then selected as the minimum distance from (0, 0) once the weights have been applied. The purpose of this procedure was to “encourage” the algorithm to select the first element in the set as the start of the sentence; however if, for example, the distance of the first saccade's start point is larger than another saccade, the smallest distance from (0, 0) will be selected to be the start point. To select the end point, the same process is applied to the saccade end points, except that the weights are reversed to “encourage” the algorithm to choose the final value. An example of this process can be seen by viewing Figure 1, Participant 1: when viewing the raw scanpath in column (a) and the processed path in column (b), it can be observed that two points are a similar distance from (0, 0). Using the weighting, the algorithm is “encouraged” to choose the earlier point as the cut-off.

The reading duration was then defined as the time between the start of the first saccade and the end of the final saccade (the rotation and the reading extraction stages are shown in Figure 1(b)). Once this was complete, any trial shorter than 500 ms or less than 2 saccades per second was excluded as it is likely the trial was of poor quality.

### 2.4. An Automated Algorithm for Classifying the Reading Eye Movements

**Figure 2:**
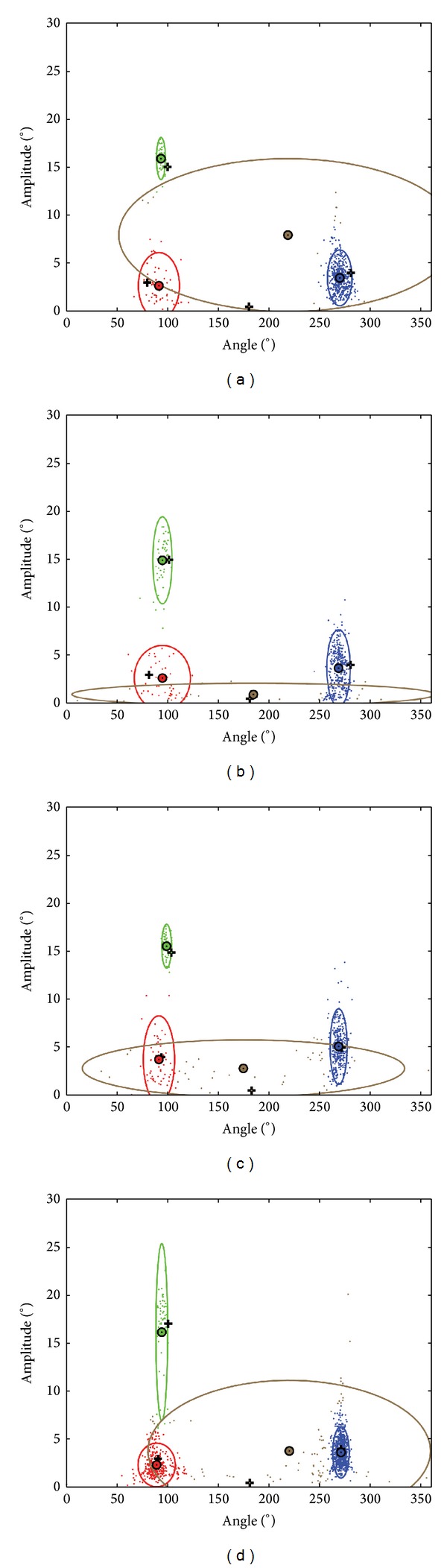
Scatterplots showing the amplitude and angle of saccades made across the 50 sentences for four examples of patients reading with the better eye. This data is used by the GMM to detect the four clusters within the data that represent the type of saccades made by the patients. The types of saccade are represented by the colours green (line change saccade), red (regression), blue (forward saccades), and brown (unknown). The black cross represents the start point for the GMM for each of the four clusters. The small circle represents the centre of the cluster and the surrounding larger ellipse represents a distribution of the data (calculated to be 2 standard deviations) captured by that cluster following the GMM process. Examples of outcomes from the GMM clustering are shown in Figure 1(c) for four different patients.

Eye-tracking software typically expresses data with general measures, such as the size (amplitude) or location of each saccade. However, in tasks such as reading, the properties of each saccade will vary according to the demands of the task. For instance, when reading, a person will make small forward saccades (from left to right). It is also common for people to “backtrack” to reread previous sections (referred to as a “regression”). The properties of a saccade occurring between the end of one line and the beginning of the line below “line change” will again differ. Finally, readers may also make saccadic eye movements that do not conform to expected patterns (unknown). For this experiment we developed an automated data analysis algorithm for classifying the types of saccade made during the task. Again we provide details of this method because it may be of use in other eye-tracking experiments. At the centre of this technique is a Gaussian mixture model that mines for clusters in the data. This approach was only possible due to the type of texts used, where line length was consistent throughout, giving predictable expected saccade angles and similar amplitudes per person. Specifically, the information needed to classify the eye movements is the amplitude (in degrees) of each saccade and the angle of each saccade, for all 50 sentences (trials) read by the “better eye” in each person. Next, it is necessary to acknowledge that the angle of eye movements occurring in a forward direction (from left to right) will occur at an* average* of 0°; for example, some forward saccades could occur at 340° and others at 20°. The discrepancy between these values, whilst indicating the same saccade type, will subsequently influence the success of the classification algorithm by yielding two separate clusters that actually give the same information. To avoid having to use circular statistics to compensate for such a scenario, we adjusted all angle values by −90°, meaning that standard statistical methods could be used (Figure 2 shows an example of this procedure in action, whereby the blue forward saccades are now located at approximately 270°). The Netlab pattern analysis toolbox [22] Gaussian Mixture Model was then used to determine four clusters with predefined start points and priors (approximate proportion of points that each cluster contains). Using this method, eye movements made by the better eye were grouped into four clusters, representing regressions, lines changes, forward saccades, and unknown saccades (Figure 2). Data yielded when reading with the worse eye was then classified in the same way, so that the proportion of saccades that fell into each of the four clusters could be calculated and compared.

### 2.5. Data Analysis

A linear mixed effects ANOVA was performed in R [23] using the linear and nonlinear mixed effects models (nlme) package to assess differences in the average reading duration and saccade rate between patients' worse and better eyes. A mixed effects model was chosen since different sentences were viewed by the worse and better eye. The random effect was set as the patient. The ANOVA was performed to test the null hypothesis, for each response, that the means for the patients' worse and better eyes are the same.

For each eye we also calculated the percentage of eye movements that were automatically classed as the four types of saccade by the classification algorithm, namely, “forward saccades,” “regressions (backwards saccades),” “between line (line change) saccades,” and “unknown” across the 50 trials read by the better and worse eye, respectively. Statistical differences in these proportions between the worse and better eye were then assessed (Wilcoxon's test).

To investigate whether the magnitudes of the change in the key measured variables between eyes for each person were important, we next calculated the difference between eyes for reading duration and saccade rate (worse eye minus better eye) to create novel “change” variables for each person. The differences between the worse and better eye were also calculated for all the measured visual function parameters (i.e., change in VF severity, VA, and CS between eyes) and then each of these resulting variables was compared to the changes in reading duration and saccade rate between eyes. Therefore, it could be determined whether larger reductions in visual field defect severity, contrast sensitivity, or visual acuity were related to a greater change in reading duration or eye movement behaviour when reading with the worse eye compared to the better eye.

Finally, differences in the median values for each of the identified eye movement types between the worse and better eye were calculated for each person; these were then compared to the change in reading duration per trial and saccade rate between eyes. Statistically significant associations were tested for using Spearman's rank correlation (rho) and also using R [23].

## 3. Results

Fourteen patients with a median age of 69 (interquartile range [IQR] of 64 to 81) years took part in the study. All participants were Caucasian and 50% were men. The patients had a range of visual field defects, visual acuity, and contrast sensitivity measures (shown in Table 1). Participants' worse eyes and better eyes were, as expected, significantly different in 24-2 MD, 10-2 MD, and PR Log CS but not in visual acuity (Wilcoxon's test). For example, median (interquartile range) between-eye difference in 24-2 MD was 9.8 (8.3 to 14.8) dB. In 10 of the 14 patients, the “worse eye” was the right eye.

Table 1 also shows median (IQR) reading durations and saccade rates for the patients' worse and better eyes. A linear mixed effects ANOVA indicated that on average patients took longer to read the sentences with their worse eye than with their better eye and this was statistically significant (*F* = 132.3, *P* < 0.001). Furthermore, patients made fewer saccades per second, on average, when reading with their worse eye compared to their better eye (*F* = 84.9, *P* < 0.001).

**Figure 3:**
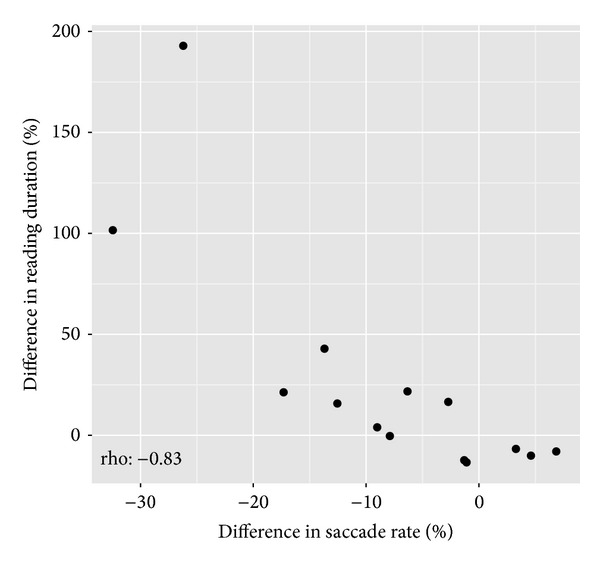
Scatterplots depicting the statistically significant relationships between the percentage difference in reading duration between the worse eye and the better eye and the percentage difference in saccade rate between the worse eye and the better eye.

When considering statistical associations for the* change *in reading duration and saccade rate between eyes, an average increase in reading duration in the worse eye compared to the better eye was closely related to an average decrease in the saccade rate in the worse eye compared to the better eye (rho: −0.83; *P* < 0.001; Figure 3). In other words, those who took longer to read with their worse eye than the better eye also had a greater reduction in saccade rate than those who read at a similar speed in each eye.

**Figure 4:**
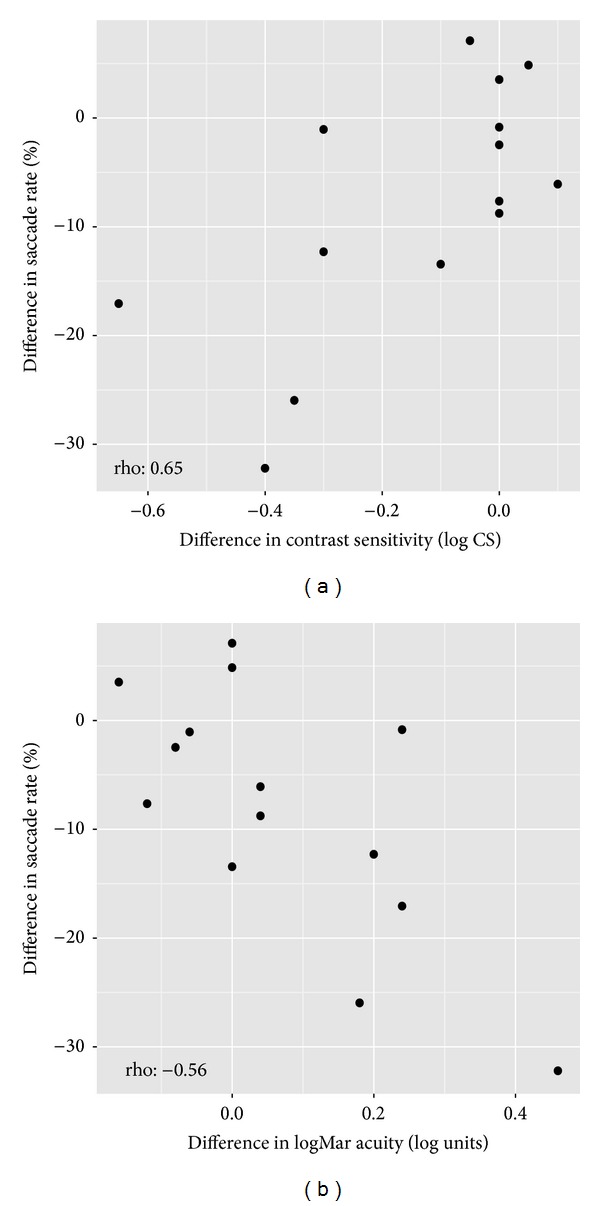
Scatterplots depicting the statistically significant relationships between (a) the difference in contrast sensitivity (log) and percentage difference in saccade rate between eyes and (b) the difference in logMAR visual acuity and the percentage difference in saccade rate between eyes.

Associations for the change in visual function measures between the better and worse eye compared with the changes for reading duration and saccade rate are shown in Table 2. There was noteworthy association between change in saccade rate and the extent of difference in contrast sensitivity between the better and worse eye. So those with a greater reduction in contrast sensitivity in the worse eye were more likely to have a reduced saccade rate in the worse eye (Figure 4(a)). Furthermore, those patients with a greater drop in visual acuity in the worse eye also showed a greater reduction in saccade rate (Figure 4(b)). There were no other statistically significant correlations (Table 2).

**Figure 5:**
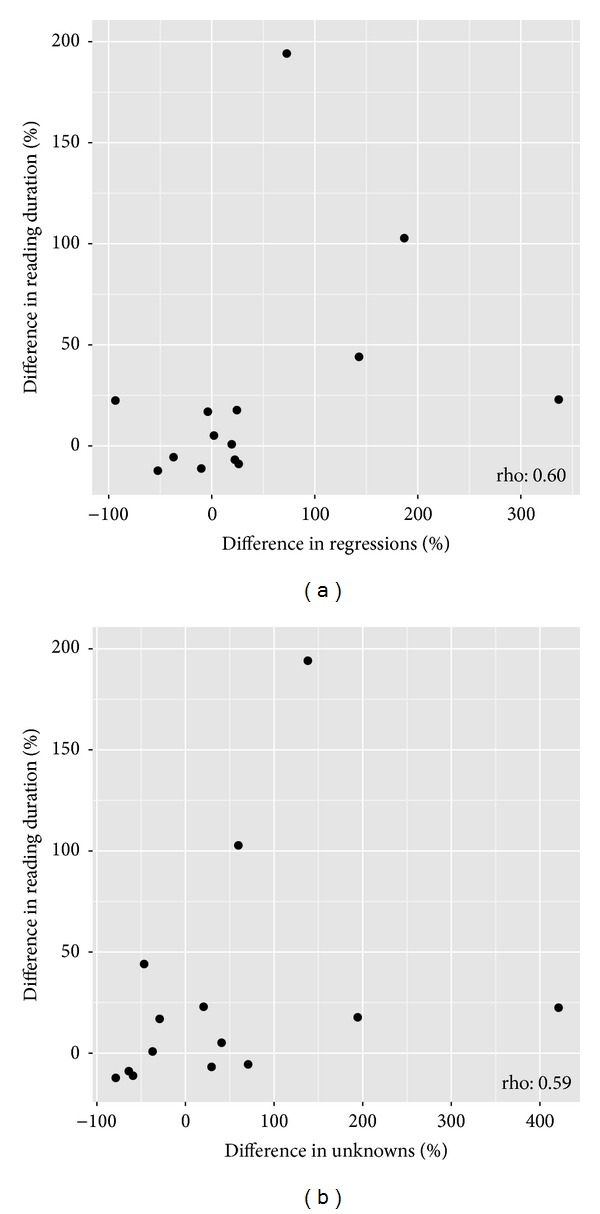
Scatterplots showing statistically significant relationships between the percentage difference in reading duration between the better and worse eye and the difference between the better and worse eye in (a) the proportion of regressions and (b) the proportion of “unknown” eye movement.

Table 3 shows the proportion of saccades classified as each of the four eye movement types for the better and worse eyes, respectively. There were no statistically significant differences in these values between eyes. However, a larger increase in reading duration in the worse eye compared to the better eye was associated with an increase in the percentage of eye movements that were regressions in the worse eye compared to the better eye (rho: 0.60; *P* < 0.03; Figure 5(a)). In addition, a greater increase in reading duration in the worse eye compared to the better eye was associated with making more unknown eye movements in the worse eye compared to the better eye (rho: 0.59; *P* < 0.03; Figure 5(b)).

## 4. Discussion

For reading, it is clear that some patients are more affected by vision loss in glaucoma than others. Some patients with glaucoma self-report difficulties with reading [6, 8, 24]. In addition, reading speed experiments indicate that patients with glaucoma have more problems with reading than people with normal vision but only “*on average*”; some patients with visual field loss performed similarly or better than people with healthy vision [9–12, 25, 26]. Reading speed can vary considerably between people making it difficult to make comparisons between patients and controls; in these studies adjustments are needed for covariates for reading speed such as education, cognitive ability, age, amount of day-to-day reading, and ethnicity. Such studies also require large sample sizes [8]. Our study examined an alternative experimental design: comparing performance between eyes in patients with asymmetrical visual field loss. Principally we demonstrated a statistically significant difference in the time it took patients to read a short passage of text in what is considered to be their worse eye (most visual field damage) when compared to their better eye (least visual field damage). This was done in a small sample of patients that carried out the reading task many times. The effect size was, however, small and the difference in reading duration between eyes was not associated with the magnitude of the difference in visual field loss between the two tested eyes. In other words, there was no “dose” effect: larger differences in severity of visual field defect between eyes were not associated with worse performance. This was true for the MD from a standard clinical visual field test (24-2 HFA) and a visual field test of more central areas (10-2 HFA). It is therefore unclear if an overall summary measure of visual field defect severity can be predictive of worsening reading performance in glaucoma. There was no significant difference between eyes for visual acuity when considering the average of all patients; this finding likely reflects the fact that many patients with worsening glaucoma maintain relatively good visual acuity while other aspects of visual function decline. However, when considering* within-person* differences in visual acuity in the worse versus the better eye, a larger decline in visual acuity was associated with a greater reduction in reading speed in the worse eye. This finding highlights the benefits of considering performance changes within each individual in addition to considering average effects across all participants. The magnitude of the difference in contrast sensitivity between eyes was also related to difference in reading performance between eyes. The important role of contrast sensitivity in reading performance in glaucoma has been emphasised elsewhere [11].

This experiment was novel in comparison with most other studies investigating reading performance in people with glaucoma because it took advantage of measurements from an eye tracker. Patients had a reduced saccade rate (making fewer saccades per second) on average when reading with their worse eye compared to their better eye. Furthermore, average saccade rate was strongly associated with reading duration. These findings imply that saccade rate, measured by an eye tracker, could be a useful surrogate for reading performance. A reduction in saccade rate in patients with visual field defects has also been observed in other studies involving different visual tasks [13, 15] and other experimental results suggest that saccadic initiation in patients with glaucoma is delayed relative to controls with healthy vision [27]. It may be that visual function loss caused by glaucoma impairs the ability of the visual system to process the surrounding information during each glance, meaning that it takes longer to initiate a saccade towards relevant information. Nevertheless, although reduced reading duration and saccade rate were observed on average for the worse eye compared to the better eye, the degree of change between eyes varied considerably across patients. For example, Figure 3 shows that certain patients had a much longer reading duration for the worse eye and also tended to show a more reduced saccade rate. However, other patients appeared to be less affected in terms of reading speed when reading with their worse eye and these people also tended to maintain a similar, or increased, saccade rate to the better eye. Typically when reading, there will be a “window” of information that can be absorbed during each fixation, referred to as the “perceptual span.” Visual degradation caused by visual field defects can be expected to reduce the number of characters that can be read with each fixation [28, 29], suggesting that more saccades must subsequently be made in order to process the same quantity of information. Therefore, some patients may have maintained an adequate reading speed when reading with their worse eye by increasing their saccade rate in order to overcome the impairment that would normally be expected due to visual degradation. This result coincides, in part, with a finding that suggests that glaucomatous visual field loss restricts saccades during other tasks such as visual search but that increasing saccade rate is associated with maintaining “good” performance [13]. It is unknown whether these eye movements are adaptive behaviour, and so this topic should be the subject of future investigation.

Eye tracking generates copious data that can be easily misidentified or misinterpreted. Eye movement analysis software for reading experiments typically provides scanpath data [21, 30, 31] that has to be manually delineated to extract specific saccades like regressions (a backtracking saccade sometimes observed during reading). So, for this study, we developed some automated techniques for identifying the different types of eye movements made during the reading task. In this experiment, there was no statistically significant difference in the types of eye movements identified by the algorithm made by the eye with more visual field damage compared to the eye with less visual field damage. Still, there was a relationship between increases in the proportion of regressions and worse reading performance. The algorithm also automatically identified unknown or “irregular” eye movements that were associated with poorer reading performance in the worse eye compared to the better eye. Patients who followed more conventional reading patterns (making a smaller proportion of regressions and unknown eye movements compared to forward saccades) in both eyes appeared to read equally quickly in both eyes. These findings illustrate the utility of eye tracking in studies of reading in glaucoma and hint at the design of future studies. For example, recent research suggests that reading performance in patients with glaucoma is particularly affected during sustained reading as opposed to when reading short passages of text [12]; it might be useful to use eye tracking in future experiments of that type.

There are limitations associated with our study. There was no assessment of comprehension of the texts and the nature of the reading experiment—large font size and reading from a computer screen—does not mimic everyday reading. The sample size was not large enough to tease out any statistically significant differences in the types of eye movements that might be used by an eye with worse visual field damage compared to one with less visual field damage. We certainly did not have enough eyes to explore how reading performance is affected by the precise location of a visual field defect or how a similar visual field defect in the right eye as compared to the left eye might influence performance; this awaits further study. Future research may also wish to consider the performance of people with asymmetric visual field loss when reading bilaterally and whether this is comparable to reading monocularly with the better or worse eye. It is also important to point out that our methods for preprocessing the eye movement data and for automatically classifying their properties have not been validated or compared with manual methods. Nevertheless, the study still adds to the literature by showing the potential of eye tracking for understanding how patients with visual field defects function in everyday tasks such as reading.

In summary, this study has shown that patients with glaucoma will take longer to read a short passage of text in what is considered to be their worse eye (most visual field damage) when compared to their better eye (least visual field damage). However, the effects were small. Unexpectedly, reading performance did not worsen in the eye with most visual field damage as the between-eye differences in visual field defect severity increased (as measured by a single summary measure of the visual field). We have also presented novel analytical eye movement data analysis that might be useful for other reading studies. The results suggest that regressions and unknown saccades result in slower reading speeds. In conclusion, we have demonstrated the utility of a novel experimental design that might help unravel the relationship between glaucomatous vision loss and difficulties with reading. For example, a future study comparing performance between eyes and using eye tracking could help determine the precise location of visual field loss that inhibits reading performance in glaucoma.

## Figures and Tables

**Table 1:** Descriptive statistics (median and interquartile range [IQR]) for key measured variables in the worse and better eye.

	Better eye	Worse eye	Wilcoxon's *P* value
24-2 MD (dB, median, and IQR)	−3.4 (−5.4, −1.8)	−14.8 (−19.5, −9.5)	<0.001
10-2 MD (dB, median, and IQR)	−3.0 (−5.0, −2.2)	−13.7 (−17.2, −9.6)	<0.001
CS (Log CS, median, and IQR)	1.85 (1.65, 1.95)	1.65 (1.38, 1.95)	0.02
VA (log units, median, and IQR)	0.11 (−0.06, 0.16)	0.13 (0.06, 0.18)	0.43
Reading duration (seconds, median, and IQR)	2.2 (1.9, 2.5)	2.4 (2.0, 2.7)	
Saccade rate (sac/sec, median, and IQR)	4.6 (4.4, 4.8)	4.3 (3.9, 4.7)	

**Table 2:** Spearman's rho correlations comparing the difference in reading duration between the worse eye and the better eye and the difference in saccade rate between the worse eye and the better eye, with key measured variables related to age and vision.

	Difference between eyes					
	24-2 MD	10-2 MD	Mean central VF points	CS	VA	Age
Change in reading duration per trial rho	−0.20	0.13	0.01	−0.41	0.35	0.17
*P* value	0.48	0.65	0.99	0.14	0.14	0.56

Change in saccade rate rho	0.19	−0.32	0.21	0.65*	−0.56*	−0.09
*P* value	0.51	0.26	0.47	0.01	0.04	0.76

Statistically significant associations are marked with an asterisk.

**Table 3:** Proportion of saccades that were forward, between lines, regressions, or unknown when reading with the best eye and worse eye, respectively.

	Better eye	Worse eye
Forward saccades (%, median, and IQR)	72.0 (70.1, 73.5)	67.2 (62.4, 75.1)
Line change (%, median, and IQR)	10.6 (9.0, 11.3)	10.2 (9.0, 11.1)
Regressions (%, median, and IQR)	11.4 (9.7, 15.4)	13.8 (10.9, 19.7)
Unknown (%, median, and IQR)	5.6 (3.7, 8.4)	6.5 (4.2, 9.8)
